# Clinical Parameters of Silent Corticotroph Adenomas With Positive and Negative Adrenocorticotropic Hormone Immunostaining: A Large Retrospective Single-Center Study of 105 Cases

**DOI:** 10.3389/fendo.2020.608691

**Published:** 2021-01-18

**Authors:** Keyi Zhang, Xuefei Shou, Hong Chen, Nidan Qiao, Wenqiang He, Zhengyuan Chen, Ming Shen, Shiqi Li, Yao Zhao, Zhaoyun Zhang, Yiming Li, Hongying Ye, Yongfei Wang

**Affiliations:** ^1^Department of Neurosurgery, Shanghai Huashan Institute of Neurological Surgery, Huashan Hospital, Shanghai Medical School, Fudan University, Shanghai, China; ^2^Department of Pathology, Huashan Hospital, Shanghai Medical School, Fudan University, Shanghai, China; ^3^Department of Endocrinology, Huashan Hospital, Shanghai Medical School, Fudan University, Shanghai, China

**Keywords:** silent corticotroph adenoma, Tpit immunostaining, refractory pituitary adenoma, non-functioning pituitary adenoma, pituitary incidentaloma

## Abstract

**Purpose:**

To investigate the different clinical characteristics of silent corticotroph adenomas (SCAs) with positive and negative adrenocorticotropic hormone (ACTH) immunostaining, and to explore the value of pituitary-restricted transcription factor (Tpit) immunostaining for diagnosing SCAs.

**Methods:**

The clinical materials of patients with SCAs who had a typical pathological feature with positive Tpit immunostaining and positive/negative ACTH immunostaining, and without clinical features and biochemical evidence for Cushing’s Syndrome in our center from April 2018 to March 2019 were analyzed retrospectively. The differences in clinical characteristics and surgical results between ACTH-positive and -negative SCAs were explored.

**Results:**

A total of one hundred and five patients (94.3% female) with SCAs were included. There were 66 SCAs with ACTH-negative (66/105, 62.9%), and 39 SCAs with ACTH-positive (39/105, 37.1%). Cases with ACTH-negative SCAs were more likely to have lower ACTH levels (27.5 ± 24.0 vs. 54.4 ± 58.6, P = 0.011), more multiple microcysts (81.8% vs. 61.5%, P = 0.022) and lower levels of Ki-67 expression (low expression rate 90.9% vs. 74.4%, P = 0.023). No statistical significant differences were observed between patients with ACTH-positive and -negative SCAs regarding gender (97.0% vs. 89.7%, P = 0.192), age (50.3 ± 10.3 vs. 49.0 ± 11.2, P = 0.543), surgical history (16.7% vs. 23.1%, P = 0.419), suprasellar extension (66.7% vs. 74.4%, P = 0.408), sphenoid sinus extension (51.5% vs. 56.4%, P = 0.627), cavernous sinus invasion (75.8% vs. 66.7%, P = 0.314), large cyst on Magnetic Resonance Imaging (MRI) (47.0% vs. 61.5%, P = 0.149), or gross total resection rate (42.4% vs. 51.3%, P = 0.379).

**Conclusions:**

ACTH-negative SCAs were observed to be more clinically silent and more likely to demonstrate multiple microcysts on MRI. The prevalence of SCAs, especially ACTH-negative SCAs, proved to be substantially underestimated and thus they should be given enough attention in consideration of the high aggressiveness of this subtype of refractory pituitary adenoma (PA).

## Introduction

Silent corticotroph adenomas (SCAs) were defined as pituitary adenomas (PAs) with positive adrenocorticotropic hormone (ACTH) immunostaining but present as clinically nonfunctioning adenomas (NFPAs) in most studies in the literature. Some SCAs are completely silent, without Cushingoid features or elevated cortisol levels. Some are clinically silent, with excess ACTH but absent of related clinical manifestations. All NFPAs lack specific clinical manifestations related to hormonal hypersecretion, including SCAs, which leads to the fact that most patients were only diagnosed and operated on when the tumor caused mass effects.

However, the 2017 World Health Organization (WHO) classification of PAs redefined a corticotroph adenoma as a PA that expresses ACTH and other proopiomelanocortin (POMC)–derived peptides and arises from adenohypophyseal cells of pituitary-restricted transcription factor (Tpit) lineage (1). With the addition of Tpit immunostaining, the criteria of SCA has changed completely. SCAs were redefined as PAs clinically diagnosed as NFPA and had typical pathological feature with positive Tpit immunostaining and positive/negative ACTH immunostaining.

The goals of this study were to investigate the different clinical characteristics between SCAs with positive and negative ACTH immunostaining and to determine the value of Tpit immunostaining for diagnosing SCAs.

## Materials and Methods

### Patients

We retrospectively reviewed the medical records of 757 cases with NFPAs who underwent pituitary surgery between April 2018 and March 2019 in our center and identified 184 patients with equivocal SCAs. A total of 105 patients with SCAs (99 women and 6 men, sex ratio 16.5:1) were included in the analysis for clinical characteristics, with a mean age of 49.8 ± 10.6 years. The inclusion criteria were: image evidence of a pituitary tumor, clinically diagnosed as NFPA, without Cushingoid features, and a typical pathological feature with positive Tpit immunostaining and positive/negative ACTH immunostaining. NFPAs are characterized by the lack of hormonal expression or secretion or any related hormonal syndrome and include a variety of pathological subtypes, as described in the latest WHO classification of PAs (1). Cases without accessible radiographs and cases without an explicit pathological diagnosis were excluded. The study was approved by the Institutional Review Board of Huashan Hospital, Shanghai Medical School, Fudan University. All patients were given informed consent when they were admitted to our center.

### Radiological Evaluation

**Figure 1:**
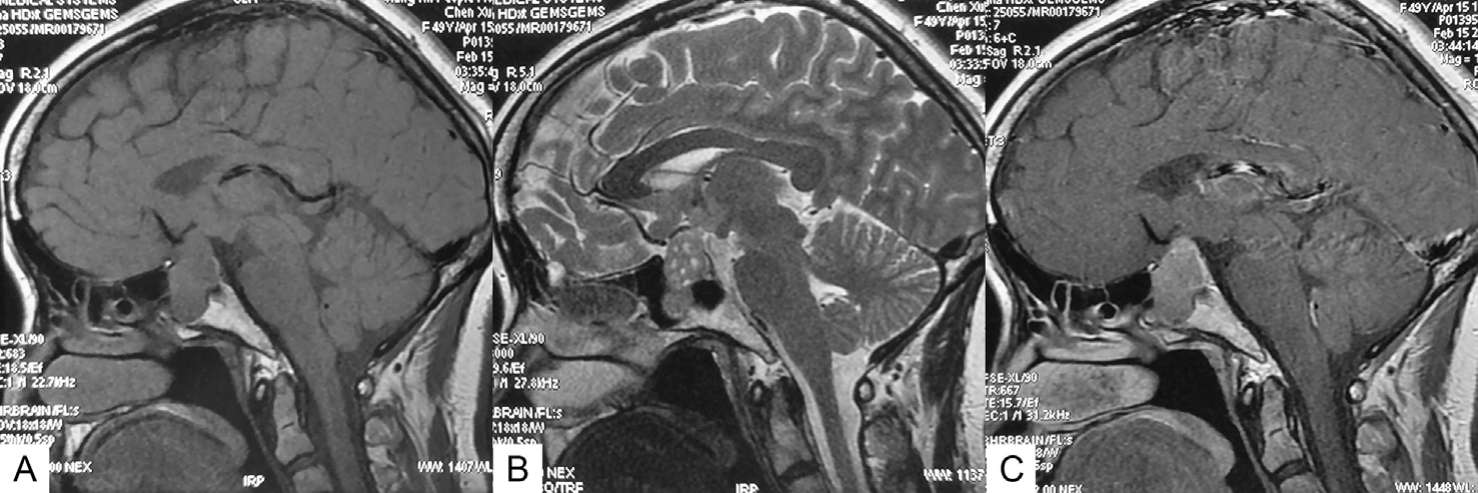
Sagittal MR images of an SCA with typical multiple microcysts. **(A)** T1-weighted image. **(B)** T2-weighted image, showing multiple microcysts. **(C)** Contrast-enhanced image.

All patients underwent conventional magnetic resonance imaging (MRI) with and without contrast before the surgery. PA smaller than 10 mm and larger than 10 mm in diameter was defined as microadenomas and macroadenomas, respectively. Suprasellar extension and sphenoid sinus extension of tumors were evaluated according to the Hardy-Wilson classification. Cavernous sinus invasion was evaluated according to the Knosp grading system. Multiple microcysts were confirmed if typical microcystic changes covered over 25% of the solid portion of the adenoma (**Figure 1**). Visual examination and visual field examination were checked in patients with radiographic compression of the optic chiasm.

### Biochemical Evaluation

A series of biochemical examinations were performed routinely on patients before the surgery to screen for hormone hypersecretion and hypopituitarism, including serum cortisol, ACTH, prolactin (PRL), testosterone or estrogen, growth hormone (GH), insulin-like growth factor-1 (IGF-1), thyroid-stimulating hormone (TSH), and thyroid hormone.

### Surgical Procedure

All operations were performed in our medical center, using either a transnasal approach or transcranial approach depending on the location and aggressiveness of the tumor (**Table 1**). To transnasal approach, intraoperative cerebrospinal fluid (CSF) leakage was evaluated according to the Kelly grading system.

**Table 1:** Different surgical approaches used for silent corticotroph adenomas (n = 105).

Surgical approach	Number (%)
Transsphenoidal	98 (93.3%)
Pterional	3 (2.9%)
Subfrontal	2 (1.9%)
Supraorbital	2 (1.9%)

### Immunohistochemical Staining

The specimen retained during the operation was fixed in 10% formalin and then gradient dehydrated by ethanol and immersed in paraffin. Section the paraffin-embedded tissue block at 2-mm thickness for immunohistochemistry. After conventional xylene and ethanol gradient dewaxing and dehydration, the sections were incubated in 3% H_2_O_2_ solution in methanol to block endogenous peroxidase activity and washed by phosphate buffer.

Subsequently, synaptophysin (Syn), ACTH, GH, PRL, TSH, follicle-stimulating hormone (FSH), luteinizing hormone (LH), Tpit, pituitary specific transcription factor-1 (PIT-1), CAM5.2, P53, SSTR2a, SF-1, and Ki-67 monoclonal antibody (Abcam Trading (Shanghai) Company Ltd., Shanghai, China) (1:50) was added, incubated at 37°C for two h and washed with phosphate buffer. Next, second antibody was applied to the slides and incubated for 30 min. Finally, the color was revealed by DAB substrate solution (Abcam Trading (Shanghai) Company Ltd., Shanghai, China) and counterstained with hematoxylin.

### Statistical Analysis

Data are reported as means standard deviations (SD) for continuous variables normally or not normally distributed, and as frequencies for categorical variables. Normality was tested using the Kolmogorov-Smirnov test. Means were compared using the unpaired t-test when data distribution was normal, or by the Wilcoxon rank-sum (Mann-Whitney) test when variables were not normally distributed. Categorical variables were compared using the chi-square test or Fisher’s exact test as appropriate. Odds ratios (ORs) and 95% confidence intervals (CIs) were calculated using univariate logistic regression. Covariates with P < 0.1 were entered into the multivariable logistic regression analysis (2). Statistical analysis was performed using the SPSS 23.0 statistical analysis software (IBM Corp., Armonk, NY). A two-tailed P value < 0.05 was considered statistically significant.

## Results

### Epidemiology

Among the 757 cases with NFPAs who underwent pituitary surgery from April 2018 to March 2019 in our center, Tpit immunostaining was performed in 66.7% of cases (505/757). We divided all cases into 2 groups based on whether Tpit immunostaining was performed. In the 505 cases with Tpit immunostaining, 138 adenomas (138/505, 27.3%) were identified as SCAs. In the 252 cases without Tpit immunostaining, 46 adenomas (46/252, 18.3%) were identified as SCAs. Therefore, the calculated proportion of SCAs in NFPAs was 24.3%.

### Clinical Features

105 patients (6 male and 99 female) with SCAs were included in the analysis for clinical parameters (**Table 2**). 33 cases with incomplete clinical data were excluded in the study. The age of the patients ranged from 17 to 74, with an average of 49.8 ± 10.6 years old. Eighty-five patients were having initial operations, and 20 patients (2 male and 18 female) had a previous pituitary surgical history.

**Table 2:** Baseline characteristics of patients (n = 105).

Characteristic	Number (%) or Mean ± S.D.
**Age**	49.8 ± 10.6
**Gender**	
Female	99 (94.3%)
**Clinical Feature**	
Vision loss or visual field defect	58 (55.2%)
Frequent headaches	24 (22.9%)
Galactorrhea, oligomenorrhoea or amenorrhea	18 (37.5%)*
Polydipsia and polyuria	4 (3.8%)
**Previous history**	
Pituitary surgery	16 (15.2%)
Pituitary surgery + radiotherapy	4 (3.8%)
**Size of tumor**	
Microadenoma	2 (1.9%)
Macroadenoma	103 (98.1%)
**Invasiveness of tumor**	
Suprasellar extension	73 (69.5%)
Sphenoid sinus invasion	56 (53.3%)
Cavernous sinus invasion	76 (72.4%)
**Cystic change of tumor**	
Large cyst	55 (52.4%)
Multiple microcysts	78 (74.3%)
**Pituitary function**	
High levels of ACTH	13 (12.4%)^†^
Hyperprolactinemia	41 (39.0%)
Central hypothyroidism	23 (21.9%)
Low levels of cortisol	19 (18.1%)
Low levels of ACTH	2 (1.9%)^†^
Low levels of testosterone	5 (83.3%)^†^
**Pathology result**	
ACTH (-), Tpit (+)	66 (62.9%)
ACTH (+), Tpit (+)	39 (37.1%)
**Ki-67 index**	
Ki-67<3%	89 (84.8%)
3%≤Ki-67<10%	16 (15.2%)
Ki-67≥10%	0 (0%)

*Only female patients of childbearing age (age 15–49) were included, n = 8.

^†^Only cases in which blood ACTH levels were tested were included, n = 81.

^‡^Only male patients were included, n = 6.

Before surgery, 58 patients (58/105, 55.2%) had vision loss or visual field defects. Twenty-four patients (24/105, 22.9%) showed frequent headaches. Among 58 female patients of childbearing age (15–49 years old), 18 (18/48, 37.5%) showed galactorrhea, oligomenorrhoea, or amenorrhea.

### Preoperative Evaluation

All 105 cases were clearly confirmed by MRI, 103 cases among which were macroadenomas, and 2 were microadenomas. Seventy-three adenomas (73/105, 69.5%) had suprasellar extension (Wilson-Hardy Grade B-C) and 56 adenomas (56/105, 53.3%) had sphenoid sinus extension (Wilson-Hardy Grade III-IV). Cavernous sinus invasion occurred in 76 cases (76/105, 72.4%) (Knosp grade 3-4). Cystic degeneration appeared in 98 cases (98/105, 93.3%), and 78 cases (78/105, 74.3%) among which were found with typical multiple microcysts.

Among all 105 cases included, hyperprolactinemia was presented in 41 patients (41/105, 39.0%). 38 patients (38/105, 36.2% had varying degrees of hypopituitarism. Twenty-four patients failed to test for serum ACTH levels due to personal reasons. In the remaining 81 cases, the serum ACTH level ranged from 2.3 to 285.6, with an average of 40.1 ± 45.5. All cases were assessed by the senior endocrinologists, confirmed with normal morning serum cortisol and no Cushingoid symptoms. Low dose dexamethasone suppression test (LDDST) was not performed preoperatively on every patient due to the limited length of hospital stay.

### Pathological Findings

**Figure 2:**
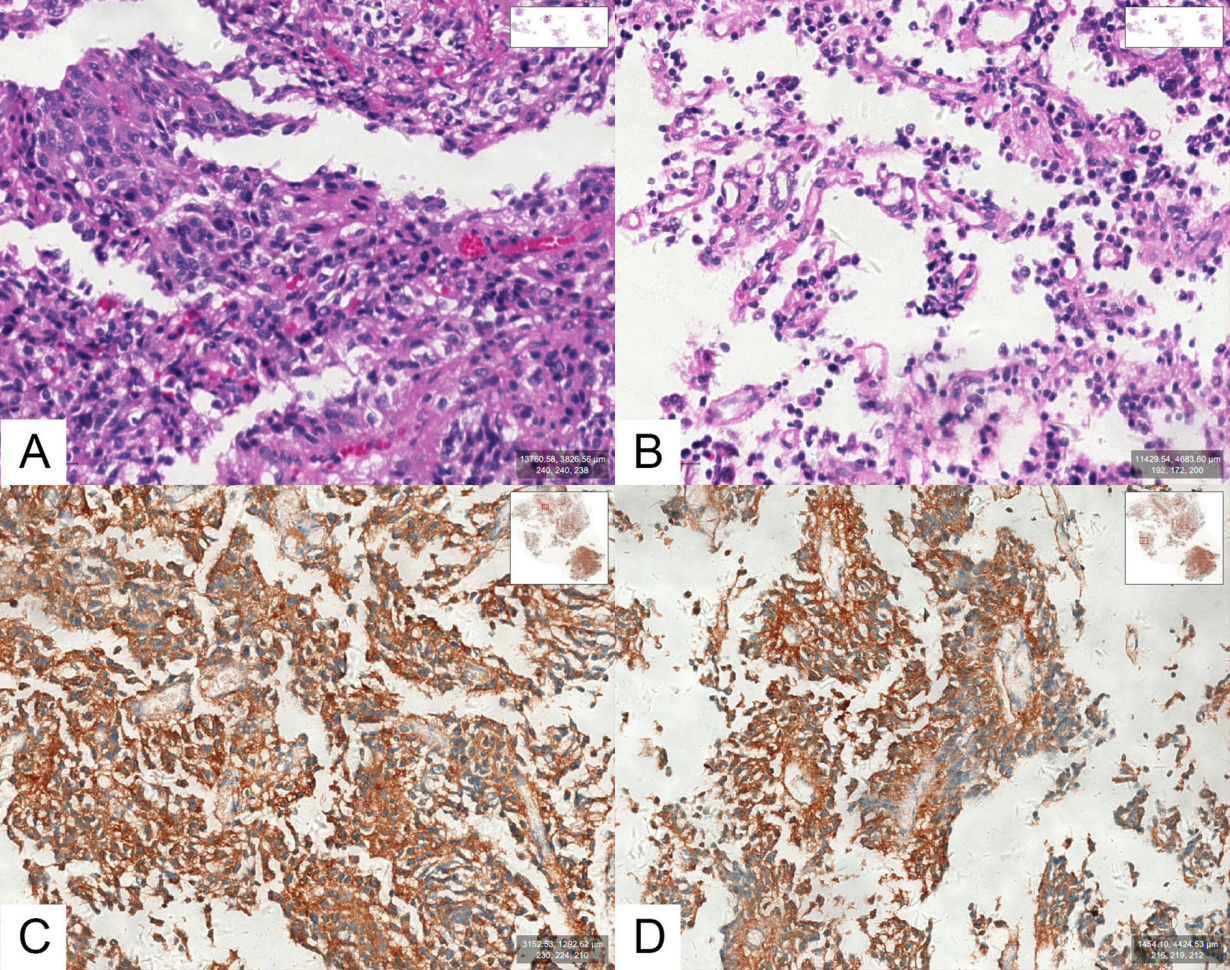
**(A, B)** Optical photographs for hematoxylin and eosin (H&E) staining in an SCA. **(C, D)** Optical photographs for ACTH staining in an SCA.

Tpit immunostaining was performed in all 105 cases, and all cases were Tpit-positive. Among them, 39 cases (39/105, 37.1%) showed positive staining for ACTH and 66 cases (66/105, 62.9%) showed negative staining for ACTH (**Figure 2**). None of the adenomas were found ACTH positive but Tpit negative in our study.

All 105 cases were stained negative for TSH, FSH, LH and PIT-1. One case was stained slightly positive for GH and PRL. Immunostaining for Syn, CAM5.2, P53, SSTR2a, and SF-1 were positive in 105 (105/105, 100.0%), 88 (88/100, 88.0%), 22 (22/103, 21.4%), 24 (24/99, 24.2%), and 3 (3/105, 2.9%) cases, respectively. Ki-67 expression was lower than 3% in 89 cases (89/105, 84.8%), between 3% and 10% (≥3%, <10%) in 16 cases (16/105, 15.2%), and no more higher than 10%.

### Surgical Outcomes

The extent of resection by microscope or endoscope were gross total resection (GTR) for 48 patients (48/105, 45.7%), subtotal resection (STR) for 50 patients (50/105, 47.6%) and partial resection (PR) for 7 patients (7/105, 6.7%). Among the 98 cases operated *via* the transnasal approach, intraoperative CSF leakage occurred in 36 cases (36/98, 36.7%). Nineteen cases (19/98, 19.4%) had grade 1 CSF leakage, 10 cases (10/98, 10.2%) had grade 2 leakage, and 7 cases (7/98, 7.1%) had grade 3 leakage.

Transient diabetes insipidus (DI) was observed in four (4/105, 3.8%) patients. Hypoadrenalism, hypothyroidism, and panhypopituitarism (≥2 endocrinological axes) occurred in 61.9% (65/105), 1.0% (1/105), and 15.2% (16/105) of all patients, respectively. An appropriate hormonal replacement was given. One patient reported decreased vision after surgery. An optic nerve decompression surgery was performed, and the patient’s vision recovered after surgery. No postoperative CSF leakage, meningitis, or permanent DI was observed.

The mean follow-up time was 17.2 ± 7.6 months (range 3-29), with three cases lost to follow-up (2 GTR, 1 STR). Forty-four patients who received GTR (44/46, 95.7%) had no recurrence at last follow-up. The 2 patients who had a recurrence both received stereotactic radiotherapy (gamma knife) immediately after recurrence were detected (3 months and 24 months after surgery, respectively).

### Group Comparison

We compared 66 (66/105, 62.9%) cases with ACTH-negative SCAs with 39 (39/105, 37.1%) cases with ACTH-positive SCAs (**Table 3**). No statistical differences were observed between the two groups regarding gender (97.0% vs. 89.7%, P = 0.192), age (50.3 ± 10.3 vs. 49.0 ± 11.2, P = 0.543), surgical history (16.7% vs. 23.1%, P = 0.419), suprasellar extension (66.7% vs. 74.4%, P = 0.408), sphenoid sinus extension (51.5% vs. 56.4%, P = 0.627), cavernous sinus invasion (75.8% vs. 66.7%, P = 0.314), large cyst on MRI (47.0% vs. 61.5%, P = 0.149) or gross total resection rate (42.4% vs. 51.3%, P = 0.379). We found that patients with ACTH-negative SCAs were more likely to have lower ACTH levels (27.5 ± 24.0 vs. 54.4 ± 58.6, P = 0.011) and multiple microcysts on MRI (81.8% vs. 61.5%, P = 0.022). ACTH-positive SCAs were more likely to have a higher level of Ki-67 expression (low expression rate 74.4% vs. 90.9%, P = 0.023).

**Table 3:** Comparison of clinical features between ACTH-negative SCAs and ACTH-positive SCAs (n = 105).

Feature	ACTH-negative (n = 66)	ACTH-positive (n = 39)	p value
Age	50.3 ± 10.3	49.0 ± 11.2	0.543
Female gender	64 (97.0%)	35 (89.7%)	0.192
Blood ACTH levels	27.5 ± 24.0^†^	54.4 ± 58.6^‡^	0.011*
Pituitary surgical history	11 (16.7%)	9 (23.1%)	0.419
Invasiveness of tumor			
Cavernous sinus invasion	50 (75.8%)	26 (66.7%)	0.314
Suprasellar extension	44 (66.7%)	29 (74.4%)	0.408
Sphenoid sinus invasion	34 (51.5%)	22 (56.4%)	0.627
Cystic change of tumor			
Large cyst	31 (47.0%)	24 (61.5%)	0.149
Multiple microcysts	54 (81.8%)	24 (61.5%)	0.022*
Gross total resection	28 (42.4%)	20 (51.3%)	0.379
Ki-67 expression (<3%)	60 (90.9%)	29 (74.4%)	0.023*

ACTH, adrenocorticotropic hormone; SCA, silent corticotroph adenoma.

*P < 0.05 was considered statistically significant.

^†^Only cases in which blood ACTH levels were tested were included, n = 43.

^‡^Only cases in which blood ACTH levels were tested were included, n = 38.

Following univariate analysis (**Table 4**), the ACTH levels, multiple microcysts on MRI, and Ki-67 expression were included in the multivariate analysis (**Table 5**). The ACTH levels (OR = 1.019; 95% CI 1.001–1.037; P = 0.036) were found as an independent factor.

**Table 4:** Univariate analysis results for the difference in clinical features between ACTH-negative SCAs and ACTH-positive SCAs (n = 105).

Feature	OR (95% CI)	p value
Age	0.988 (0.952–1.026)	0.539
Female gender	0.273 (0.048–1.568)	0.146
Blood ACTH levels	1.020 (1.003–1.039)	0.025*
Pituitary surgical history	1.500 (0.559–4.024)	0.421
Cavernous sinus invasion	0.640 (0.268–1.530)	0.316
Suprasellar extension	1.450 (0.600–3.504)	0.409
Sphenoid sinus invasion	1.218 (0.549–2.700)	0.627
Large cyst	1.806 (0.807–4.045)	0.150
Multiple microcysts	0.356 (0.145–0.873)	0.024*
Gross total resection	0.700 (0.316–1.551)	0.379
Ki-67 expression (<3%)	3.448 (1.142–10.410)	0.028*

ACTH, adrenocorticotropic hormone; SCA, silent corticotroph adenoma; OR, odds ratio; CI, confidence interval.

*P < 0.05 was considered statistically significant.

**Table 5:** Multivariate analysis results for the difference in clinical features between ACTH-negative SCAs and ACTH-positive SCAs (n = 105).

Feature	OR (95% CI)	p value
Blood ACTH levels	1.019 (1.001–1.037)	0.036*
Multiple microcysts	0.378 (0.130–1.096)	0.073
Ki-67 expression (<3%)	3.528 (0.907–13.730)	0.069

ACTH, adrenocorticotropic hormone; SCA, silent corticotroph adenoma; OR, odds ratio; CI, confidence interval.

*P < 0.05 was considered statistically significant.

## Discussion

In this study, we have identified the different manifestations between ACTH-positive and ACTH-negative SCAs and calculated the proportion of SCA in all NFPAs based on whether Tpit immunostaining was performed. Most previous studies about SCAs had no ACTH-negative cases included, and in existing researches about ACTH-negative SCAs, the numbers of cases were rather smaller. 2017 WHO classification of PAs defined SCA as a subtype of PA that belongs to the TPIT lineage (1) and Tpit immunostaining was suggested to be added to the diagnostic protocol (3). After Tpit immunostaining was added, the diagnostic rate of SCA in all NFPAs improved significantly. Our results suggested that without performing Tpit immunostaining, up to 62.9% of cases (66/105) could be missed, which proved the necessity to give more attention to this substantially underestimated subtype of PA.

In our study, serum ACTH levels were found significantly higher in ACTH-positive SCAs (54.4 ± 58.6 vs. 27.5 ± 24.0., P = 0.011) than in ACTH-negative SCAs, and clearly above the normal range. In previous studies, most series found that SCAs (only ACTH-positive cases included) had elevated ACTH levels and normal cortisol levels (4, 5), which agrees with our findings. Several hypotheses have been proposed to explain the lack of clinical hypercortisolism in SCAs despite the high expression of ACTH in ACTH-positive SCAs. However, recent studies about cell origin and molecular mechanisms still need further experiments to be proven. In ACTH-negative SCAs, the serum ACTH levels were mostly normal (average ± std.). Because the commercially available ACTH antibodies cannot separate bioactive or bio-inactive ACTH from pro-ACTH or POMC (6), presumably none of the three is elevated in the circulation of patients with ACTH-negative SCAs. Thus, ACTH-negative SCAs were completely clinically SCAs, which present with no hypersecretion of bioactive ACTH, bio-inactive ACTH, pro-ACTH, or POMC.

We observed that multiple microcysts appeared more often in ACTH-negative SCAs (81.8% vs. 61.5%, P = 0.022) than in ACTH-positive cases. According to the literature, more cysts were observed in SCAs (only ACTH-positive cases included) than NFPAs, with 75% of them showing multiple microcysts, which agrees with our results (61.5% of ACTH-positive SCAs and 81.8% of ACTH-negative SCAs showed multiple microcysts) (7, 8). However, this imaging manifestation also appeared in most null cell adenomas and gonadotroph adenomas according to some series (8). In other words, ACTH-negative SCAs and null cell adenomas were similar in lack of hormone secretion and imaging findings, though differ greatly in recurrence rate (9).

In order to check and predict the possible SCAs, several studies have investigated the difference in baseline characteristics between SCAs and NFPAs. SCAs were found more prevalent in females compared with NFPAs in most series (3, 7, 10, 11), while the male-female ratio in our study was 1:16.5; Age at presentation was mostly similar in patients with SCAs and NFPAs (7, 10, 12–14). Patients with SCAs (only ACTH-positive cases included) secreted ACTH higher than normal (4, 5, 10). SCAs were found with more microcysts and intratumoral hemorrhage than NFPAs (7, 10, 12, 15). Cavernous sinus invasion observed in SCAs compared to NFPAs was either higher or similar in the literature (3, 4, 7, 14, 16). Thus, female gender, high blood ACTH levels (only for ACTH-positive SCAs), and cystic degeneration were found as relatively reliable predictors for SCAs, and the predictive significance of tumor invasiveness remains to be confirmed.

Possible mechanisms of the “silence” feature of SCAs include two main hypotheses. Kovacs et al. first introduced this type of PA in 1978 and put forward a hypothesis that ACTH was not discharged from the adenoma cells due to abnormal lysosomal function or abnormal hormone synthesis (17). Another hypothesis suggested that the low expression level of prohormone convertase (PC) 1/3 in SCAs caused the lack of post-transcriptional regulation of POMC, which led to the silence feature of SCAs (18–20). Moreover, we also found that in a patient who had a pituitary surgical history, the ACTH immunostaining was positive for the previous operation and negative for the current operation, which needs further research to be explained.

Limitations in this study included: 1) We did not perform a comprehensive endocrine assessment for every patient before the surgery. Considering almost all of the cases in our study (103/105) were nonfunctioning pituitary macroadenomas, and that the Cushingoid symptoms of cases with ACTH-secreting pituitary macroadenomas can be considerably serious, the possibility of Cushing’s Disease can be roughly ruled out. 2) The follow-up period was relatively short for estimating the long-term recurrence rate in our series. Most of our patients come from other cities outside Shanghai. It is difficult for them to visit us regularly after surgery if they were feeling no sign of recurrence.

## Conclusions

The ACTH-negative SCAs appeared to be more clinically silent, and more likely to show multiple microcysts on MRI. The addition of Tpit immunostaining significantly improved the diagnostic rate of SCA. The prevalence of SCA was substantially underestimated, and sufficient attention should be paid to this high-risk, aggressive type of refractory PA.

## Data Availability Statement

The original contributions presented in the study are included in the article/supplementary material. Further inquiries can be directed to the corresponding authors.

## Ethics Statement

The protocol was approved by the Huashan Hospital, Shanghai Medical School, Fudan University. All subjects gave written informed consent in accordance with the Declaration of Helsinki.

## Author Contributions

KZ, XS, HY, and YW contributed conception and design of the study. KZ organized the database and performed the statistical analysis. KZ and XS wrote the first draft of the manuscript. HC, NQ, WH, and ZC contributed to the acquisition of data for the work. MS, SL, YZ, ZZ, YL, HY, and YW revised the work critically for important intellectual content. All authors contributed to the article and approved the submitted version.

## Funding

The present work was supported by the Science and Technology Commission of Shanghai Municipality (Grant no. 17441901400). The present work was supported by the National Natural Science Foundation of China (Grant no. 81602191).

## Conflict of Interest

The authors declare that the research was conducted in the absence of any commercial or financial relationships that could be construed as a potential conflict of interest.
